# Beyond the Limits: tRNA Array Units in *Mycobacterium* Genomes

**DOI:** 10.3389/fmicb.2018.01042

**Published:** 2018-05-17

**Authors:** Sergio M. Morgado, Ana C. P. Vicente

**Affiliations:** Laboratory of Molecular Genetics of Microorganisms, Oswaldo Cruz Institute, Oswaldo Cruz Foundation, Rio de Janeiro, Brazil

**Keywords:** mycobacteria, tRNA array units, code:Perl, mycobacteriophages, mycobacteria plasmids, tmRNA and structured RNA, bioinformatics, HNH endonuclease

## Abstract

tRNA array unit, a genomic region presenting an intriguing high tRNA gene number and density, was supposed to occur only in few bacteria phyla, particularly *Firmicutes*. Here, we identified and characterized an abundance and diversity of tRNA array units in *Mycobacterium* associated genomes. These genomes comprised chromosome, bacteriophages and plasmids from mycobacteria. Firstly, we had identified 32 tRNA genes organized in an array unit within a mycobacteria plasmid genome and therefore, we hypothesized the presence of such structures in *Mycobacterium* genus. However, at the time, bioinformatics tools only predict tRNA genes, not characterizing their arrangement as arrays. In order to test our hypothesis, we developed and applied an *in-house* Perl script that identified tRNA genes organization as an array unit. This survey included a total of 7,670 complete and drafts genomes of *Mycobacterium* genus, 4312 mycobacteriophage genomes and 40 mycobacteria plasmids. We showed that tRNA array units are abundant in genomes associated to the *Mycobacterium* genus, mainly in *Mycobacterium abscessus* complex species, being spread in chromosome, prophage, and plasmid genomes. Moreover, other non-coding RNA species (tmRNA and structured RNA) were also identified in these regions. Our results revealed that tRNA array units are not restrict, as previously assumed, to few bacteria phyla and genomes being present in one of the most diverse bacteria genus. We also provide a bioinformatics tool that allows further exploration of this issue in huge genomic databases. The presence of tRNA array units in plasmids and bacteriophages, associated with horizontal gene transfer, and in a bacteria genus that explores diverse niches, are indicatives that tRNA array units have impact in the bacteria biology.

## Introduction

Transfer RNAs (tRNAs) are smalls non-coding RNAs with the main function associated to the translation machinery and, therefore, essential for all living organism. Besides this role, tRNAs were associated with other functions such as signaling and regulatory molecules in diverse cellular processes (Raina and Ibba, 2014). The number of tRNA genes is highly variable among organisms (Marck and Grosjean, 2002), but in bacteria, considering only the non-redundant isoacceptor types, the number of tRNA genes varies from 28 to 44. In bacteria, the tRNA gene number has no bias to fast or slow growing; high or low GC content and intracellular or free living species. However, when the total number of tRNA genes is taken into account, a remarkable increase (e.g., twice/three times) might be observed even in species within genus, e.g., *Bacillus, Bacteroides, Lactobacillus, Listeria, Oceanobacillus, Photorhabdus*, and *Shewanella* (Bailly-Bechet et al., 2007). In general, the tRNA genes are found dispersed in the genomes but they also are encoded in small clusters containing two to five tRNAs. This later organization is particularly common in mitochondrial genomes (Jung et al., 2010; Friedrich et al., 2012; Li et al., 2014) and in the *Entamoeba* genus. In some species from this genus, there are multiple tandem arrays of clustered tRNA genes containing up to five tRNA genes (Tawari et al., 2008). Contrasting with the regular organization and distribution of tRNA genes in the genomes, large tRNA gene clusters, presenting high number and density (i.e., tRNA/kb), have been observed in some prokaryote and bacteriophage genomes (Green and Vold, 1993; Bailly-Bechet et al., 2007; Hatfull et al., 2010; Pope et al., 2011, 2014; Puerto-Galán and Vioque, 2012; Delesalle et al., 2016; Alamos et al., 2017). Concerning the presence of large tRNA clusters in bacteria, a recent large-scale genome survey characterized an abundance of tRNA array units mainly in *Firmicutes* and few other low GC Gram-positive bacteria phyla. In high GC content phyla, as *Actinobacteria*, they were found only in few genomes but none from *Mycobacterium* genus (Tran et al., 2016).

Interestingly, analyzing the genome sequence of a *Mycobacterium* sp. (CBMA231 strain), recovered from a Brazilian soil, we identified a plasmid (~270 kb) harboring a tRNA array unit composed by 32 tRNAs. This finding and the occurrence of tRNA arrays in the cluster M mycobacteriophages (Pope et al., 2014) raised our hypothesis of the occurrence of this element in mycobacteria genomes.

Currently, there are several software that predict tRNA genes in nucleotide sequences (e.g., ARAGORN, ARWEN, tRNAscan-SE) but their genomic organization in arrays is not characterized. Therefore, in order to test our hypothesis, we developed a Perl script to identify tRNA array units and applied it in a large-scale complete/draft genome survey associated to the *Mycobacterium* genus. This strategy allowed accessing a large data set encompassing a broad mycobacteria genomes spectrum (chromosomes, mycobacteriophages, and plasmids). Our findings suggest that tRNA array units are abundant in genomes associated to the *Mycobacterium* genus, being spread in chromosome, prophage and plasmid genomes. These arrays could be characterized in multiple groups according to their tRNA gene isotype organization and besides, RNA species other than tRNA were also identified in such structures.

## Materials and methods

### Genomes analyzed

Complete and draft genomes (*n* = 7670) of *Mycobacterium* genus available in June 2017 were obtained from National Center for Biotechnology Information (NCBI) ftp site (ftp://ftp.ncbi.nlm.nih.gov/genomes/genbank/bacteria). Incomplete draft genomes were filtered out from the data set. Mycobacteriophage genomes (*n* = 4312) were obtained from NCBI and Actinobacteriophage Database (http://phagesdb.org/). Mycobacteria plasmids were also used in the analysis, including those deposited in NCBI database and pCBMA213_1 from *Mycobacterium* sp. CBMA213 (data not shown), a isolate recovered from the Atlantic Forest soil, sequenced using Nextera paired-end library on Illumina Hiseq 2500 (Oswaldo Cruz Foundation high-throughput platform) and deposited in the Bacteria Collection of Environment and Health (CBAS, Fiocruz). The sequenced plasmid sequence is deposited in Genbank under access number: MF600313.

### tRNA gene prediction, identification, and classification of tRNA arrays

The tRNA gene prediction of the data set was performed by ARAGORN v1.2.37 (Laslett and Canback, 2004) using bacterial genetic code. These predictions were confirmed using other tRNA gene prediction software, tRNA-scan (Lowe and Eddy, 1997). The output files generated by ARAGORN (using parameter -fons) were used as input files in an *in-house* script to the identification of tRNA array units (available in Supplementary Data Sheet 1). Each tRNA predicted by ARAGORN provides information about its genomic coordinate and contig number, which were accessed by our *in-house* script. tRNA array units were defined as genomic regions with 20 or more tRNAs and a minimum density of two tRNA/kb (Tran et al., 2016). The developed script identifies tRNA array units, initially, defining a search window with the minimum number of tRNAs (i.e., 20), in a same contig, storing the genomic coordinates of the first and last tRNA. With this information is possible to calculate the length and the density of the putative array. If the tRNA density of the genomic region with the minimum number of tRNAs (i.e., 20) is lower than that stipulated (i.e., 2 tRNAs/kb), the search window advances a position relative to the first and last tRNA, and the tRNA density is recalculated. In a genomic region with the minimum number of tRNAs, if tRNA density is ≥ 2 tRNAs/kb, then the region comprehending the first and last tRNA is considered as a partial array. Next, the search window advances a position relative only to the last tRNA, increasing the number of tRNAs in the array, and next, tRNA density is recalculated. If tRNA density keeps ≥ 2 tRNAs/kb, then this new region is considered the current partial array. If the increasing of the search window results in a region with tRNA density <2 tRNAs/kb, then the partial array registered early is considered the final array. The process of increasing the search window is repeated until all predicted tRNAs are analyzed. tRNA Array Finder script generates as output a tab-delimited file in which each line provides information about each genome analyzed: total number of tRNAs in the genome; number of tRNAs in the array; tRNA array length; genomic coordinate (begin/end) of the array; tRNA array density; and the number of the contig containing the array. If a genome contains more than one tRNA array unit, each the new one is registered on the next line with a progressive counting (i.e., #1, #2, #3…). The tRNA array units were classified in groups according to their tRNA gene isotype synteny (using the single-letter amino acid code abbreviation). Although the chosen representation to show the organization of the isotypes was an amino acid alignment, the gaps (– symbol) may not represent the actual distance between two adjacent tRNA genes, but the distance from the reference array. The *Acidithiobacillus ferrooxidans* ATCC 23270 genome was used as positive control to the identification of tRNA array unit (Tran et al., 2016).

### Genetic analysis

To determine the genetic relationship among the genomes containing tRNA arrays, we performed a Multilocus sequence analysis (MLSA) based on seven concatenated housekeeping genes (16S rRNA, *sec*A, *gyr*B, *fus*A, *tuf*, *hsp*65, and *rpo*B) totaling ~14.3 kb. Reference and representative mycobacteria genomes were included in this analysis. To observe the genetic relationships of the tRNA genes from the arrays, their nucleotide sequence were retrieved and concatenated, totaling ~5.3 kb. All the concatenated sequences were submitted to Maximum-likelihood analysis with the GTR substitution model and 100 bootstrap replications using PhyML V3.1 (Guindon et al., 2010). All alignments were performed by MAFFT v7.271 (Katoh and Standley, 2013). The tree figures were edited using iTOL (Letunic and Bork, 2016).

### Identification of orthologous clusters, sequence annotation, plasmid, and phage identification

Gene content analysis of the tRNA arrays regions were done, initially, by extraction of nucleotide sequences of the arrays and their flanking regions (2 kb). These sequences were annotated using Prokka v1.12 (Seemann, 2014) and submitted to orthologues clustering and identification by GET_HOMOLOGUES v3.0.5 (Contreras-Moreira and Vinuesa, 2013) using parameters of minimum coverage of >= 70% and identity >= 40%.

From the genomes containing tRNA arrays, the corresponding contig with the array was extracted and used to plasmid and phage identification. Mycobacteria plasmid markers, *rep*A and relaxase (Ummels et al., 2014) had their sequences used as query to blastn searches. The putative plasmid contigs identified were also checked manually and submitted to blastn on NCBI to comparative analysis with known mycobacteria plasmids. Phage identification was performed by PHASTER (Arndt et al., 2016) using those contigs with tRNA array units as input.

## Results

### Identification and characterization of tRNA arrays in chromosomes, prophages, and plasmids of mycobacteria

A total of 7,670 complete and drafts genomes of *Mycobacterium* genus, 4312 mycobacteriophage genomes and 40 plasmids were surveyed for the presence of tRNA gene. The regular set of housekeeping tRNA genes were identified in all mycobacteria genomes, however some genomes harbor a higher number of tRNA genes (*n* > 70) in comparison with the average observed in this bacteria genus (n~50). Moreover, an unusual presence of tRNA genes (*n* = 1–36) were detected in some mycobacteriophage genomes and mycobacteria plasmids.

In order to identify the presence of any tRNA array unit in these genomes we used our *in-house* script that was based on the tRNA array unit definition: a genomic region containing at least 20 tRNA genes with a minimal density of two tRNA genes per kilobase (Tran et al., 2016). The survey identified a total of 367 tRNA arrays within 7670 mycobacteria genomes, most of them (*n* = 362) containing one array and five with two tRNA arrays each. Besides, 102/4312 mycobacteriophage genomes and 2/40 plasmids presented tRNA arrays.

The tRNA array units identified in the mycobacteria chromosomal genomes contained from 20 to 39 tRNAs genes with 53% harboring 36 tRNA genes, these array units encompassed 3–19 kb regions resulting in densities ranging from 2 to 9 tRNAs/kb (Supplementary Table S1). The mycobacteriophage arrays contained 21–29 tRNA genes, most have 27 tRNA genes (61/102) distributed in 4.4–7.3 kb region with a density of ~4–6 tRNA/kb (Supplementary Table S2). The mycobacteria plasmids carrying tRNA arrays units were recovered from *Mycobacterium chimaera* (*n* = 2), besides *Mycobacterium* sp. CBMA213 plasmid (this study) and in putative plasmids identified in *Mycobacterium chelonae* (*n* = 1) and *Mycobacterium abscesssus* (*n* = 4) draft genomes (this study). These arrays contained 26–37 tRNAs genes encompassed in ~3–12 kb regions with densities of 2–9 tRNAs/kb (Supplementary Table S3).

### tRNA array distribution among *Mycobacterium* species

Our analysis revealed the presence of tRNA array units in at least 13/154 *Mycobacterium* species so far described, including fast- and slow-growing species as well clinical and environmental strains. The majority of genomes (315/362) belong to *Mycobacterium abscessus* complex (fast-growing species with the highest number of genomes available), including *Mycobacterium massiliense* and *Mycobacterium bolletii* subspecies. Other fast growing species also harboring tRNA array are: *M. chelonae* (*n* = 8), *Mycobacterium immunogenum* (*n* = 3), *Mycobacterium saopaulense* (*n* = 3), *Mycobacterium mageritense* (*n* = 2), *Mycobacterium conceptionense* (*n* = 2), *Mycobacterium canariasense* (*n* = 2), *M*.sp. (*n* = 12), *Mycobacterium austroafricanum, Mycobacterium fortuitum*, and *Mycobacterium wolinskyi*. Four slow-growing species were identified carrying tRNA array units: *Mycobacterium avium* (*n* = 7), *M. chimaera* (*n* = 2), *Mycobacterium bouchedurhonense*, and *Mycobacterium koreense*.

### Types of tRNA array

**Figure 1:**
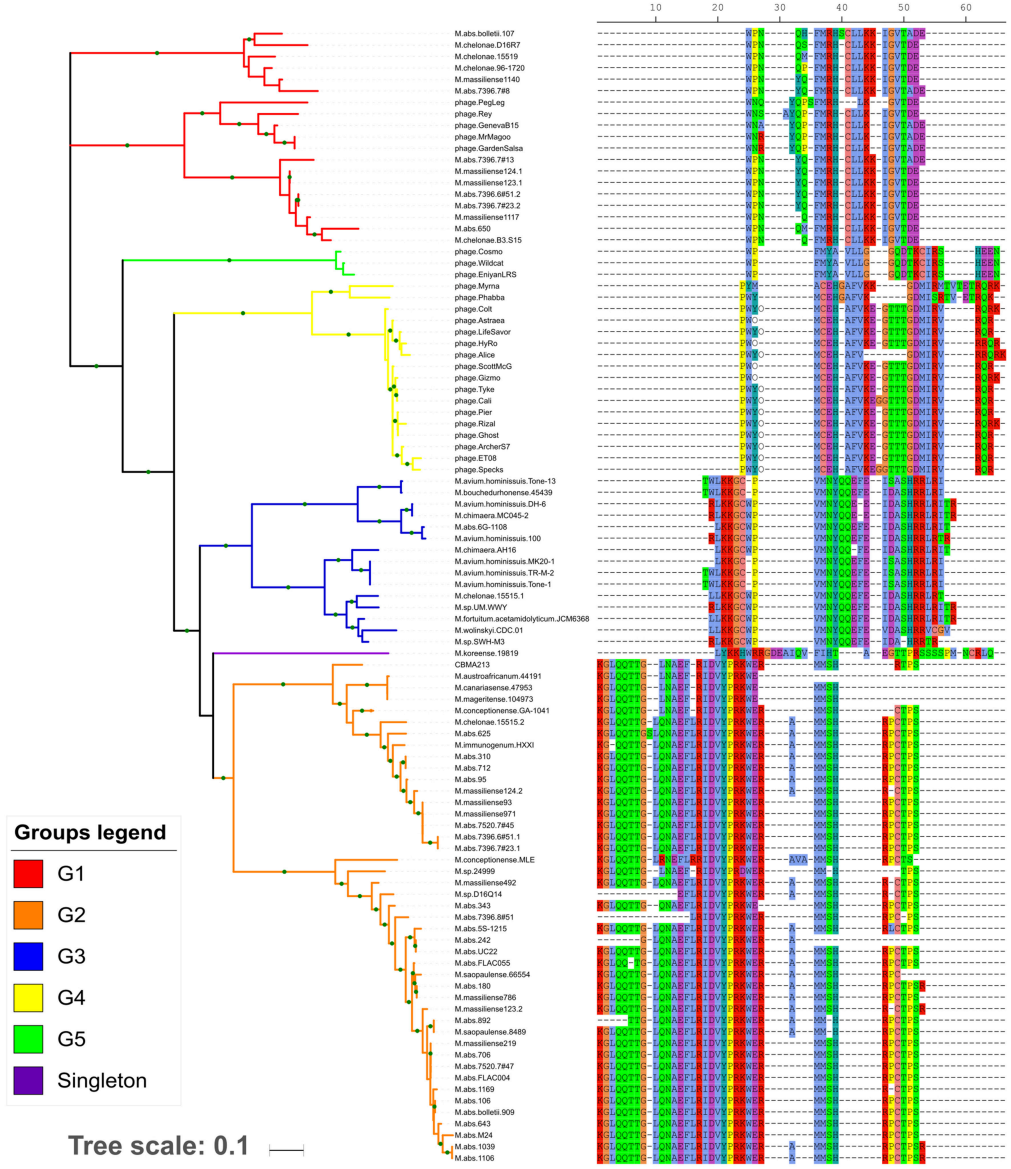
Maximum likelihood tree based on concatenated tRNA gene nucleotide sequences. In the right side, the tRNA isotype organization (using the single-letter amino acid code) is related with each tree branch. The gaps (– symbol) may not represent the actual distance between two adjacent tRNA genes, but the distance from the reference array. Each array group is depicted by the branch color. The green circles in the branches indicate bootstrap values ≥70.

Tran et al. (2016), based on the tRNA amino acid isotypes and organization of each array, assigned the arrays in seven groups. Following these criteria, we could discriminate five new groups (G1-G5) and a singleton unique to *Mycobacterium* genus (Figure 1 and Supplementary Table S4) genera.

The G2 group was the most prevalent and diverse, presenting high number of variants (Supplementary Table S5) spread in at least eight fast-growing mycobacteria species and two/eight plasmids. The G3 group was present in both slow- and fast-growing *Mycobacterium* species (*n* = 3 and *n* = 4, respectively) as well in six plasmids. The G1 group was restricted to fast-growing *M. abscessus* complex species and *M. chelonae*, besides cluster M mycobacteriophages. The G4 and G5 groups were exclusively found in cluster C and V mycobacteriophages, respectively. The singleton corresponded to an array only present in *M. koreense*. Although the tRNA amino acid isotypes are conserved within the groups, their gene sequences present some variability (Figure 1).

Overall, the 20 amino acid isotypes were represented in the six new array types. Within some groups, it was observed slightly variants that eventually lacked and/or duplicated some isotype species (Supplementary Table S5). Comparing the tRNA isotypes present in the arrayed and non-arrayed (whole genome) regions, it was observed an isotype repertoire redundancy. However, for genomes from some few lineages (67 genomes, Supplementary Table S6), it was observed an increment of the isoacceptors species (one or two per genome, 12 non-redundant isoacceptors in total). Interestingly, in the repertoire of G4 group, which contains the tRNA array present in the mycobacteriophage from cluster C, besides the 20 standard amino acid isotypes, there were the two unusual genetically-encoded amino acids: selenocysteine and pyrrolysine. The later was also present in the tRNA arrays from some genomes of a *M. abscessus* lineage (876, 877, 878, and 887 genomes).

### Genomic features of tRNA arrays

Besides tRNAs, other non-coding RNA species were also identified in the arrays of chromosomes, plasmids and mycobacteriophages. One copy of tmRNA gene was within tRNA array present in several mycobacteria and mycobacteriophage genomes. Most of the arrays that contain tmRNA gene were in mycobacteriophage genomes (Supplementary Table S4). Genetic analysis showed that the tmRNA gene sequences were highly similar within G4 group, contrasting with the sequences from G1 group (Supplementary Figure S1). Other non-coding RNA species identified was a structured RNA ~430 nucleotide length, which is present within ~ 81% of the arrays, being prevalent in G1 and G2 groups, and absent in G3 group.

Several CDS (Coding DNA Sequence) were identified as part of the arrays, most of them coding hypothetical proteins. The analyses of these CDS revealed an overall intra and inter gene content diversity among the groups. The gene content comparison among the tRNA array types did not reveal any common gene among all of them, but an endonuclease coding sequence (often identified as homing endonuclease HNH family) was the most widespread CDS among the groups (Supplementary Figure S2). Considering the G1 array gene content, within the mycobacteria genomes and mycobacteriophages, there was a core represented by one CDS (hypothetical gene with RF-1 domain) and two CDS (HNH endonuclease and hypothetical one), respectively. G2 group, the most widespread and diverse among mycobacteria genomes, harbor from two to 20 genes being the XRE regulator and HNH endonuclease genes presented in ~87% of the arrays. G3 group harbor from one to nine genes with a ubiquitous gene which encodes an endonuclease annotated as CRISPR-associated endonuclease Cas9. G4 group (cluster C mycobacteriophages) presented one to seven genes, being four genes shared by ~96% of the tRNA array units. Two of them were hypothetical, but one encoded a Peptidyl-tRNA hydrolase (PTH2-like family) and a beta-lactamase domain protein (transpeptidase superfamily). G5 group presented a core with two hypothetical genes and some arrays harbor more one to two hypothetical genes. In the singleton tRNA array there were two CDS coding for an endonuclease and a hypothetical protein.

We determined the GC content of the tRNA array group regions since a discrepancy between their GC content and the host could be evidence to their acquisition by horizontal gene transfer. The GC content varied 62–68%, in the mycobacteria chromosome; 61–64%, in the plasmids; and 56–65%, in the bacteriophages. In general, the GC content of the tRNA array and the host were quite similar. The tRNA arrays from G1 and G5 group present the lowest GC% even considering distinct host (mycobacteria species and mycobacteriophages; Supplementary Table S4).

Considering the overall gene content of the plasmids harboring the tRNA array units (G2 and G3 groups) it was identified plasmids core genes represented by six genes, including HNH endonuclease, type VII secretion-associated serine protease mycosin, AAA family ATPase, helicase, replicative DNA helicase and a CDS coding for a hypothetical protein. All plasmids presented the same replication system characterized by the *rep* gene, except pCBMA213_1.

### tRNA array unit flanking regions

In order to enlarge our knowledge about the genomic context of these units we extended the analysis of the array to its flanking regions. In general, no common features were identified among these regions considering the five groups. Most of the CDS coded for non-redundant hypothetical proteins, it was not identified any signs of recent integrative events. However, some CDS, localized two/three CDS upstream the first tRNA from the array units, coded for endonucleases, what could represent a vestige of an integrative event (Burt and Koufopanou, 2004).

We extend the analyses to search for the presence of prophages and we found that in some contigs containing tRNA array units from mycobacteria genomes there were traces of, or intact prophages, flanking or encompassing the tRNA array units. These features were only identified in G1 tRNA array group (Supplementary Table S7). In addition, the most common prophage identified corresponded to the Pegleg phage from cluster M mycobacteriophages.

### tRNA array genomic epidemiology

In order to get insights on the origin, evolution, and spread of the tRNA array units within their hosts we performed a phylogenetic analysis based on mycobacteria MLSA scheme correlating with the tRNA array groups carried by the genomes. These genomes are from distinct mycobacteria species isolated from all continents, most clinical strains, and the tRNA arrays here identified were located in distinct genomic compartments (chromosome/plasmid/*Mycobacterium* prophage; Supplementary Table S4).

**Figure 2:**
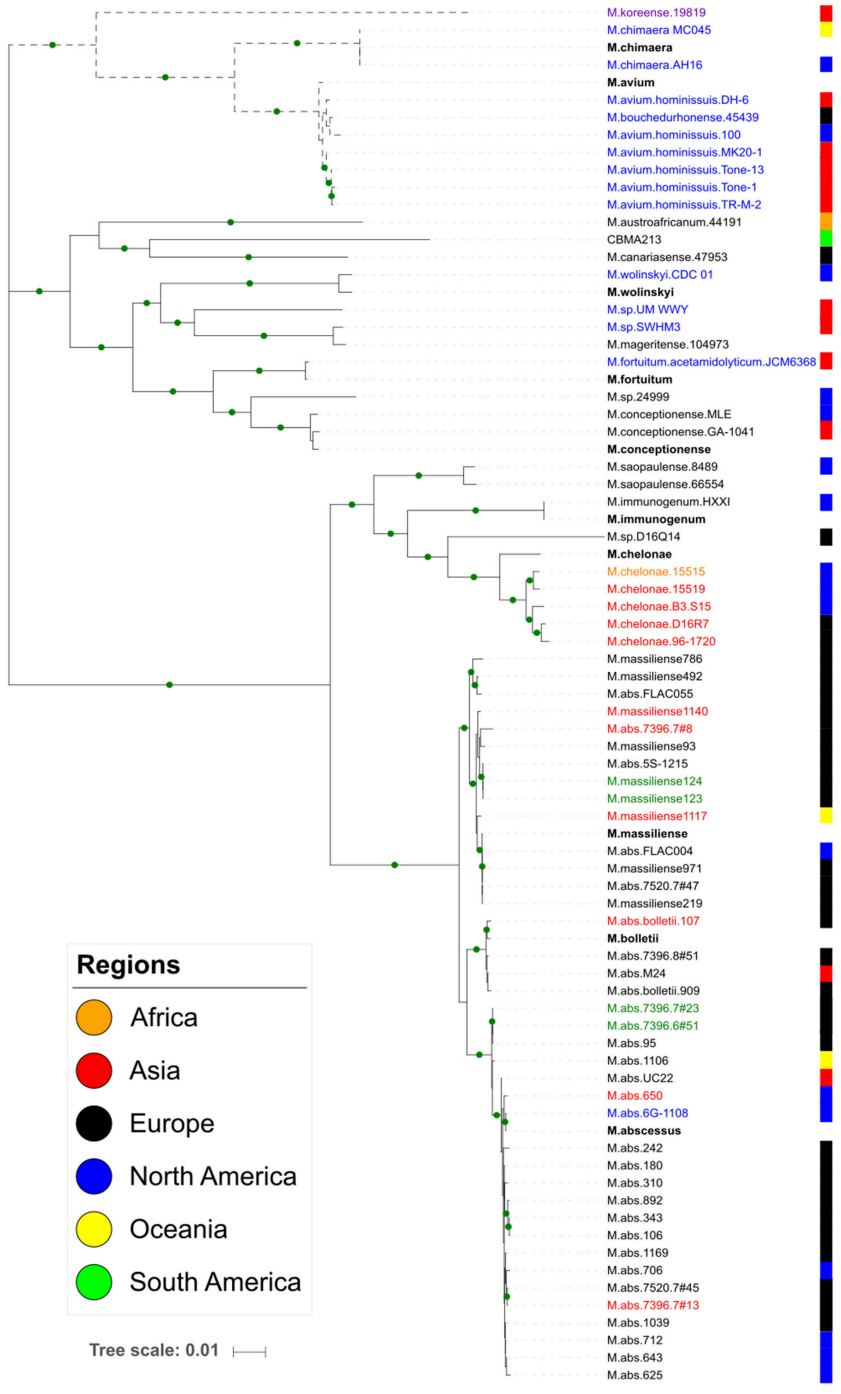
Maximum likelihood tree based on seven concatenated housekeeping genes. The green circles in the branches indicate bootstrap values ≥70. Each colored block at the right indicates the geographic location of the strain isolation. Dotted branches represent the slow-growing species. The array group is indicated by the species name label: G1, red; G2, black; G3, blue; Singleton, lilac; both G1 and G2, green; both G2 and G3, orange. The reference species are in bold.

The MLSA phylogeny, based on the mycobacteria genomes, determined four major clusters I-IV (Figure 2). The cluster I is the only one comprehending slow-growing species (*M. koreense, M. chimaera, M. avium*, and its subspecies). The tRNA arrays from this cluster are associated to their chromosome, except in the *M. chimaera* strains, where they are in plasmids. Interestingly, one *M. chimaera* strains is environmental/Australia and the other clinical/USA. All other strains are clinical from Japan, EUA, France and South Korea. The cluster II comprehends a diverse group of closely related species and all of them but CBMA213 strain harbor its tRNA array in the chromosome. The CBMA213 is an environmental strain from Brazil, which carries a plasmid of ~270 kb where the tRNA array is located. The other strains are from China, India, Japan, Malaysia, Spain, United Kingdom, and USA. The cluster III harbors *M. saopaulense* (USA), *M. immunogenum* (USA), and *M. chelonae* (Belgium, Germany and USA) from clinical and environment. Most of these genomes are from *M. chelonae* strains with tRNA arrays located in their chromosome or *Mycobacterium* prophage genome. Besides, one *M. chelonae* has two tRNA arrays, one in a plasmid and other in the chromosome. The *M. abscessus* complex, including *M. massiliense* and *M. bolletii* subspecies, forms the cluster IV with all strains being clinical and most with tRNA array in the chromosome. In this cluster some tRNA arrays are associated to *Mycobacterium* prophages and one is in a plasmid.

In order to get insights on the origin of the tRNA array units we performed a genetic analysis based on the tRNA isotypes vs. the genomes where they were found. It was demonstrated the distribution of the same tRNA group (G1–G5) in distinct genomic context (chromosome, prophage and phage genome, plasmid). The G1 tRNA array was found in *M. abscessus* and *M. chelonae* chromosomes and some prophages but also in cluster M mycobacteriophages. The G2 tRNA array was also characterized in *M. abscessus* and *M. chelonae* besides other species and, in general, associated to chromosomes. Moreover, this array group was identified in two distinct plasmids, one from *M. chelonae* 15515 clinical/USA and the other *M*.sp. CBMA213 environmental/Brazil. The G3 tRNA arrays were within chromosome of slow- and fast-growing species, except three located in plasmids from *M. abscessus* and *M. chimaera*. Interestingly, so far, the G4 and G5 tRNA arrays were exclusively found in genomes from cluster C and V mycobacteriophages, respectively (Figure 1).

## Discussion

In the current study, we have comprehensively exploited genomes associated to the *Mycobacterium* genus using computational algorithms to characterize tRNA array units, providing the first scenario of its distribution, localization and composition in this genus from *Actinobacteria* phyla. The algorithm developed to identify tRNA array units was used in association with the software ARAGORN. Currently, the available bioinformatics tools predict only tRNA genes, but not characterize their arrangement in arrays. Even thought, the role of tRNA array units in the bacteria biology is not clearly understood, some authors argue their importance to fast growing bacteria, as in *Escherichia coli* (Ardell and Kirsebom, 2005), and the coordination of tRNA transcription vs. translation rates (Rudner et al., 1993). By the other hand, a unique study showed they do not induce apparent phenotypic differences when deleted from a carrier organism (Puerto-Galán and Vioque, 2012). Therefore, the availability of a bioinformatics tool which allows the screening of tRNA arrays in a broad range of genomes might reveal their prevalence among organisms, raising the real scenario of the presence of such structures and further inference on their role in the organisms biology.

Using mycobacteria genomes as model, we showed the potential of this tool in tRNA array characterization showing the number of tRNA genes and the array density and length. In a previous study, using *in house* scripts, tRNA array units had been surveyed and identified in a set of complete genomes, but they were not made available (Tran et al., 2016). Moreover, no tRNA array units were identified among the complete mycobacteria genomes considered in their study. Contrasting with their result, we showed that tRNA array units are abundant in this genus being found in chromosome and/or plasmid of several species, including fast- and slow-growing species. Besides, tRNA array units were characterized also in mycobacteriophage genomes. In fact, tRNA islands had already been observed in a set of clinical strains of *M. abscessus* complex and *Mycobacterium sp*, from Malaysia (Choo et al., 2014; Wee et al., 2016). Our results revealed that the tRNA arrays seems to be disseminated among genomes associated to this genus whereas we characterized them in at least 13 recognized *Mycobacterium* species, besides 12 genomes from species without designation. Furthermore, 102 mycobacteriophage genomes, belonging to three distinct clusters (C, M, and V), and five distinct plasmids, from four *Mycobacterium* species, also contained this kind of element. Tran et al. (2016) analyzed a set of *Actinobacteria* complete genomes and did not observe any tRNA array in genomes from *Mycobacterium* genus. Probably, these results were due to the limitation of genomes and species considered at the time of analysis (2013). For instance, a complete genome of *M. abscessus* (UC22 strain), deposited in 2015, was here identified as positive to the presence of a tRNA array. Considering the mycobacteriophage genomes, Pope et al. (2014) had already characterized tRNA clusters in cluster M mycobacteriophages. Some other studies mentioned the presence of a high number of tRNA genes in mycobacteriophages from clusters C and V (Hatfull et al., 2010; Delesalle et al., 2016), but they did not characterize their organization as tRNA clusters. Here we corroborated the presence of the arrays in cluster M and revealed also the clusterization of tRNA genes in mycobacteriophages from cluster C and V. We also revealed the presence of tRNA arrays in other mycobacteria mobilome compartment, including five phylogenetic non-related plasmids ranging from ~195 to 437 kb and harboring 26 to 37 tRNA genes.

The tRNA isotypes present in the five groups are redundant in relation to the ones in non-arrayed regions. However, 12 new isoacceptor species are in arrays harbored by some mycobacteria species genomes, one of them corresponds to the amino acid pyrrolysine, which is decoded and synthesized by a limited number of organisms and not yet reported in this bacteria genus (Prat et al., 2012) The availability of new isoacceptor species could reflect in the bacteria fitness, affecting fast-, and slow-growing mycobacteria species.

Based on comparative array organization, it was hypothesized that they were transfer from *Firmicutes* or unknown donor to reduced taxonomic clades (Tran et al., 2016). Supporting the lateral gene transfer hypothesis of these elements, we have some evidences from M*ycobacterium* genus: (i) the presence of a same array group, defined by the synteny of the tRNA isotypes, in not closely related mycobacteria species and in association with genomes of mobile elements such as mycobacteriophages and plasmids; (ii) prophages harboring tRNA array units that are embedded in some mycobacteria genomes; (iii) the dispersion of strains from a same lineage in several environments while keeping the tRNA array unit; (iv) the presence of a same array group in plasmids harbored by different *Mycobacterium* species. Furthermore, G1 array, characteristic of cluster M mycobacteriophages, was identified in some mycobacteria chromosomes, and both compartments presented a quite similar GC content. Analysis by PHASTER showed that some G1 tRNA array regions of mycobacteria genomes contain archeological traces or are embedded in cluster M *Mycobacterium* prophages, being Pegleg phage the most common. These evidences support the hypothesis that tRNA array units have been transferred from mycobacteriophages to mycobacteria genomes, at least for G1 group. Indeed, cluster M mycobacteriophages harbor an integrase gene (Delesalle et al., 2016), which would have allowed their integration into the mycobacteria genome and further acquisition by the bacteria lineage. Mycobacteriophages act as vehicles for intein gene dissemination (Kelley et al., 2016), and in a similar manner, they could act in the tRNA arrays dissemination in mycobacteria. In fact, the presence of tRNA gene clusters in phage genomes would be unexpected since bacteriophages are supposed to depend completely on the translation apparatus of the hosts (Kunisawa, 2000; Bailly-Bechet et al., 2007). However, the presence of tRNA genes in bacteriophages could be explained based on codon/amino acid usage, which would increase the fitness of viral infection and the host range (Bailly-Bechet et al., 2007; Delesalle et al., 2016). Similarly, tRNA arrays from G2 and G3 groups were identified in plasmids and in several mycobacteria species, suggesting that plasmids would be other vehicle in arrays dissemination.

Since the tRNA isotypes from the arrays are redundant compared with those in non-arrayed regions, we compared the nucleotide sequence of all tRNA genes in order to find out whether the arrays were resulted from recent intra-genomic duplication events (identity ≥90%). Considering the 20–39 tRNA genes in the arrays, only 1 (majority) to 3 tRNA genes showed high identity to tRNA genes present in the other genomic regions, contrasting with the majority of genes which present low or no identity (data not shown). It suggests that only few tRNA genes from the large arrays could be involved in an eventual duplication events. Therefore, considering the tRNA arrays as units (composed by 20 or more tRNA genes), these evidences do not support the hypothesis of the tRNA arrays being originated from intra-genomic duplication events. This evidence, in addition to the presence of a tRNA array with same isotype synteny in different species and genomes (mycobacteriophages and plasmids), support the hypothesis of their horizontal transfer.

Among the coding genes identified in the genetic context of the arrays, endonucleases, mainly HNH homing endonucleases, were the most widespread. In *Mycobacterium* species, some homing endonucleases are abundant, such as intein-associated endonucleases (Burt and Koufopanou, 2004). In the arrays from cluster M mycobacteriophages, adjacent HNH endonucleases were implicated in the generation of tRNA repertoire diversity (Pope et al., 2014), and in the same manner, they could be implicated to the other arrays presenting endonucleases. Curiously, genes related to ribosome rescue and translation machinery were also identified in some arrays from G1 and G4 groups. Among them, the release factor 1 (RF-1), peptidyl-tRNA hydrolase (Pth), and tmRNA genes (Singh and Varshney, 2004; Janssen and Hayes, 2012). Since tRNA arrays would provide more tRNA species to the bacteria, this sudden increase could affect the functioning of the ribosomes, thus extra rescue factors could be necessary.

This analysis based on the current *in-house* script confirmed our hypothesis of the tRNA array existence in mycobacteria genomes conversely to the previous scenario of their prevalence, particularly in *Firmicutes*. The abundance of tRNA arrays within distinct mycobacteria genomes suggests that such structures may have different roles in the distinct hosts. The bioinformatics tool described here enables the exploration of tRNA arrays in prokaryote, archaea, and eukaryote genomes. Further large studies are needed to provide evidences on the role and the impact of these structures in the biology of the organisms.

## Author contributions

AV and SM designed and wrote the manuscript; SM analyzed the data.

### Conflict of interest statement

The authors declare that the research was conducted in the absence of any commercial or financial relationships that could be construed as a potential conflict of interest.
