# Supplementary material for: Beyond the Limits: tRNA Array Units in Mycobacterium Genomes
Source: Front Microbiol. 2018 May 17;9:1042. doi: 10.3389/fmicb.2018.01042 (PMC5966550; doi:10.3389/fmicb.2018.01042)
Supplement: Supplementary file 5 [file Table_5.DOC]

**Supplementary Table S5.** tRNA array groups based on their isotype organization.

| **tRNA array groups** | **tRNA array variants** |
| --- | --- |
| **1** | WP--NQH-FMRHSCLLKKIGVTADE  WP--NYQ-FMRH-CLLKKIGVTADE  WP--NYQ-FMRH-CLLKKIGVT-DE  WP--NQM-FMRH-CLLKKIGVT-DE  WP--NQS-FMRH-CLLKKIGVT-DE  WP--NQP-FMRH-CLLKKIGVT-DE  WN-RYQP-FMRH-CLL-KIGVTADE  WN-AYQP-FMRH-CLL-KIGVTADE  WNSAYQP-FMRH-CLL-KIGVT-DE  WN-QYQPSFMRH---LK--GVT-DE |
| **2** | KG-LQQTTG-LQNAEFL-RIDVYPRKWER--AMMSHRPCTPSR  KG-LQQTTGSLQNAEFL-RIDVYPRKWER--AMMSHRPCTPSR  KG-LQQTTG-LQNAEFL-RIDVYPRKWER--AMMSHR-CTPSR  KG-LQQTTG-LQNAEFL-RIDVYPRKWER--AMMSHRPCTPS-  KG-LQQTTG-LQNAEFL-RIDVYPRKWER--AMMSHRLCTPS-  KG-LQQTTG-LQNAEFL-RIDVYPRKWER--AMMSHR-CTPS-  KG-LQQTTGSLQNAEFL-RIDVYPRKWER--AMMSHRPCTPS-  KG-LQQTTGSLQNAEFL-RIDVYPRKWEO--AMMSHR-CTPS-  KG-LQQTTG-LQNAEFL-RIDVYPRKWER--AMMSHRPCTP--  KG-LQQTTG-LQNAEFL-RIDVYPRKWER--AMMSHRPC----  KG--QQTTG-LQNAEFL-RIDVYPRKWER--AMMSHRPCTPS-  KG-LQQT-G-LQNAEFL-RIDVYPRKWER--AMMSHRPCTPS-  KG-LQQTTG-LQNAEFL-RIDVYPRKWER---MMSHRPCTPS-  KG-LQQTTG-LQNAEFL-RIDVYPRKWER---MMSHR-CTPS-  KG-LQQTTG-LQNAEFL-RIDVYPRKWER---MMSHRP-----  ------TTG-LQNAEFL-RIDVYPRKWER--AMM-HRPCTPS-  KG-LQQTTG-LQNAEFL-RIDVYPRKWER--AMM-HRPCTPS-  KG-LQ-TTG-LQNAEFL-RIDVYPRKWER--AMMSHRPCTPSS  --------------EFL-RIDVYPRKWER--AMMSHR-CTPS-  KG-LQQTTG--QNAEFL-RIDVYPRKWE----MMSHRPCTPS-  KG-LQQTTGSLQNAEFL-RIDVYPRKWEOE-AMMSHR-C-PS-  ----------------L-RIDVYPRKWER---MMSHRPC-PS-  --------G-LQNAEFL-RIDVYPRKWER--A-----------  KGTLQQTTG-LQNAEFL-RIDVYPRKWER--AMMSHR-CSL--  KG-LQQTTG-L-NAEF--RIDVYPRKWE----MMSH-------  KG-LQQTTG-L-NAEF--RIDVYPRKWE---------------  KG-LQQTTG-L-NAEF--RIDVYPRKWER---MMSH--RTPS-  KG-LQQTTG-L-NAEF--RIDVYPRKWER---MMSH--CTPS-  KG-LQQTTG-L-NAEF--RIDVYPRDWER---MMH----TPS-  KG-LQQTTG-L-NAEF--RIDVYPRDWER---MMH--------  KG-LQQTGS-LQNAEFL-RI-VYPRKWER---M--HR-CTPS-  KG-LQQTTG-LRN-EFLRRIDVYPRKWERAVAMMSHRPCTS-- |
| **3** | --LKKGCWPVMNYQQEFEIDASHRRLRIT-  -LLKKGCWPVMNYQQEFEIDASHRRLRITR  -RLKKGCWPVMNYQQEFEIDASHRRLRITR  -LLKKGCWPVMNYQQEFEIDASHRRLRT--  -RLKKGCWPVMNYQQEFEIDASHRRLRTR-  --LKKGCWPVMNYQQ-FEIDASHRRLRIT-  -RLKKGCWPVMNYQQE-EIDASHRRLRITR  -LLKKGCWPVMNYQQEFEVDASHRRVCGV-  -RLKKGCWPVMNYQQEFEIDA-HRRTR---  TWLKKG--PVMNYQQEFEISASHRRLRI--  TWLKKGC-PVMNYQQEFEISASHRRLRI--  --LKKGC-PVMNYQQEFEISASHRRLRI--  TWLKKGC-PVMNYQQEFEIDASHRRLRI-- |
| **4** | PWYOMCEH-AFVKE-GTTTGDMIRV-----RQR--  PWYOMCEH-AFVKE-GTTTGDMIRV-----RQRK-  PWYOMCEH-AFVKEGGTTTGDMIRV-----RQR--  PW-OMCEH-AFVKE-GTTTGDMIRV-----RQR--  PW-OMCEH-AFVKE-GTTTGDMIRV-----RQRK-  PW-OMCEH-AFVKE-GTTTGDMIRV-----RRQR-  PW-OMCEH-AFVKEGGTTTGDMIRV-----RQRK-  PW-OMCEH-AFVKEGGTTTGDMIRV-----RQR--  PWYOMCEH-AFV-------GDMIRV-----RRQRK  PWYOMCEH-A---------GDMIRV-----RRQRK  PY-MACEHGAFVKK-----GDMIRMTVTETRQRK-  PW-YMCEHGAFVK------GDMISRTV-ETRQK-- |

The tRNA isotype organization of each array is represented using the single-letter amino acid code and colored according to iTOL scheme (Letunic and Bork, 2016). Although the representation chosen to show the organization of the isotypes was an amino acid alignment, the gaps (- symbol) may not represent the actual distance between two adjacent tRNA genes, but the distance from the reference array.

Letunic, I., and Bork, P. (2016). Interactive tree of life (iTOL) v3: an online tool for the display and annotation of phylogenetic and other trees. *Nucleic Acids Research*, *44*(Web Server issue), W242–W245. http://doi.org/10.1093/nar/gkw290
