# Supplementary figures and images for: Beyond the Limits: tRNA Array Units in Mycobacterium Genomes
Source: Front Microbiol. 2018 May 17;9:1042. doi: 10.3389/fmicb.2018.01042 (PMC5966550; doi:10.3389/fmicb.2018.01042)

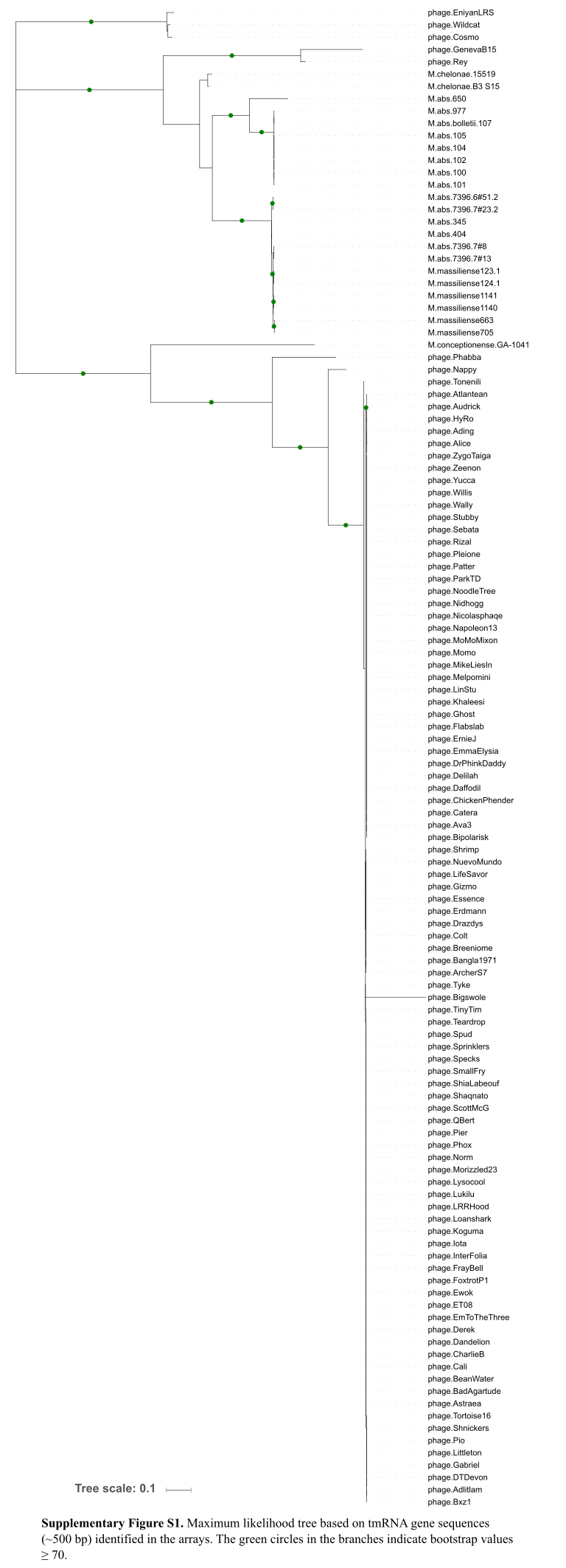

Supplement: Supplementary file 8 [file Image_1.JPEG]

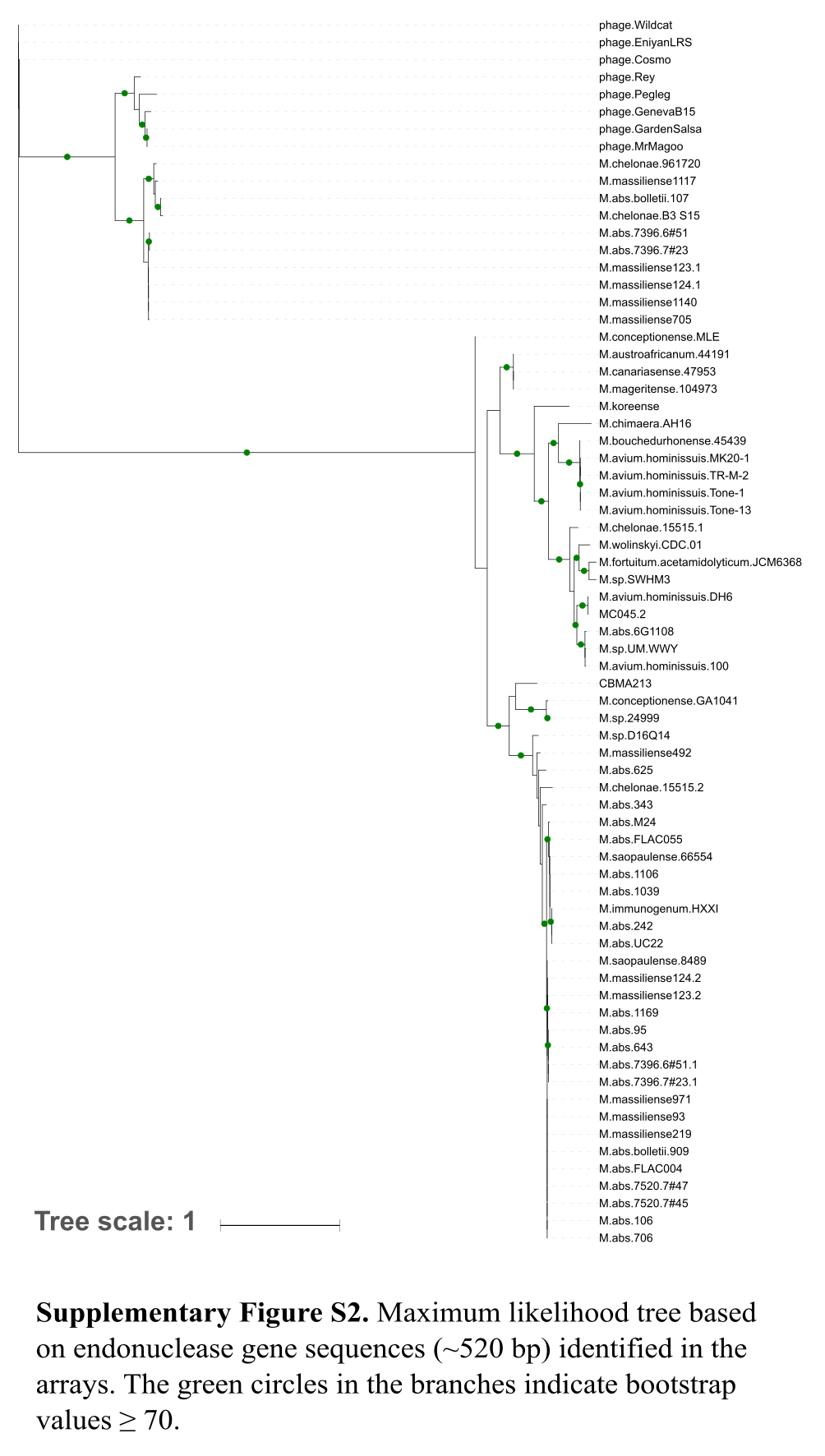

Supplement: Supplementary file 9 [file Image_2.JPEG]
